# Improving electrocoagulation performance by adding environmentally friendly materials

**DOI:** 10.1038/s41598-025-14462-6

**Published:** 2025-09-12

**Authors:** Manal M. M. Barakat, Mona S. S. Soliman, Mahmoud F. Mubarak

**Affiliations:** 1 Central Laboratory for Environmental Quality Monitoring (CLEQM), National Water Research Center (NWRC), El-Kanater, P.O. Box 13621/6, Qalubiya, Cairo Egypt; 2 Petroleum Application Department, Egyptian Petroleum Research Institute (EPRI), Cairo, 11727 Egypt; 3 Corelabcenter, Egyptian Petroleum Research Institute (EPRI), 1 Ahmed El Zomor St., Nasr City, Cairo 11727 Egypt

**Keywords:** Electrocoagulation, Taro mucilage, Wastewater treatment, Iron/aluminum electrodes, Kinetics studies, Electrochemistry, Environmental chemistry, Physical chemistry, Theoretical chemistry

## Abstract

This study investigates the enhancement of electrocoagulation (EC) treatment of wastewater by adding mucilage extracted from Egyptian taro (*Colocasia esculenta*). EC is an efficient, eco-friendly method for removing pollutants such as total dissolved solids, total suspended solids (TSS), color, and chemical oxygen demand (COD) from wastewater. The research explores the effects of different operational parameters, including applied voltage, treatment time, and pH, on the efficiency of EC using iron/aluminum electrodes. Mucilage was added as an environmentally friendly additive to enhance removal efficiency. The study used a laboratory-scale setup with kinetic and thermodynamic analyses to evaluate the removal mechanisms. Results indicate that the addition of mucilage improved COD removal, while other parameters like color and TSS were better without additives. The findings demonstrate that mucilage can be a potential enhancer in wastewater treatment, with the Langmuir and Freundlich models applied to describe the adsorption process.

## Introduction

The considerable increase in pollution of water resources, coupled with the dramatic rise in population and urbanization, has led to a significant increase in the concentration of both organic and inorganic contaminants^1^. Domestic, agricultural, and industrial effluents are the primary sources of this pollution, creating severe challenges for the environment and aquatic life. In response to this growing issue, the reuse of wastewater has become an essential requirement. To address water pollution, a variety of treatment techniques have been developed, including ion exchange, adsorption, physical, chemical, and biological methods, as well as ultrafiltration, reverse osmosis, chemical precipitation, and electrocoagulation^2^.

Among these, there is a pressing need to develop cost-effective, efficient, and environmentally friendly technologies for hydrogen gas production, with Electrolysis standing out as a promising method^3^. Electrolysis involves the passage of an electric current through a solution containing undesired ions, facilitating a non-spontaneous chemical reaction. In addition to its application in hydrogen production, electrochemical technology is increasingly viewed as a viable solution for the degradation of organic pollutants in water. Electrochemical processes such as electro-oxidation, electro-flocculation, electro-reduction, electro-deposition, and electro-coagulation offer significant potential for addressing water pollution in a sustainable manner^1,4^.

Electrocoagulation (EC) is an electrochemical process widely used in wastewater treatment due to its ability to rapidly eliminate a broad range of pollutants, including organic compounds, suspended particles, turbidity, color, and even heavy metals^5^. This versatility makes EC shows potential for improving wastewater treatment. The underlying mechanism of electrocoagulation involves an oxidation/reduction reaction, which occurs within an electrochemical cell. In this cell, sacrificial electrodes connected to a power source are immersed in the wastewater to be treated. The difference in electric potentials between the electrodes leads to the generation of flocs, followed by coagulants that help remove undesired species from the water in the form of metal hydroxides^6,7^.

During the EC process, ions are continuously generated through the electrochemical decomposition of the sacrificial anode. These ions dissolve in water, forming various coagulant species, including metallic hydroxide precipitates and other metal ion complexes. Simultaneously, hydrogen gas bubbles are produced at the cathode due to water electrolysis. The process of electrocoagulation typically occurs in three stages: (a) destabilization of contaminants, (b) aggregation of charged particles into flocs, and (c) oxidation of electrolytic species to form coagulants. The hydrogen bubbles play a crucial role in the flotation of these flocs, aiding in their separation from the treated water. Electrodes used in EC, commonly made from aluminum or iron, offer several advantages, including availability, low cost, non-toxicity, and high oxidation states. These properties, along with the flotation effect of hydrogen bubbles, enhance pollutant removal efficiency, making EC an effective wastewater treatment solution^8–10^.

When the electrochemical cell starts working and a potential was been applied through the electrodes, the anode and cathode electrodes undergo oxidation and reduction reactions, respectively. Equations (1–3) summarize the reactions taking places at the metal electrodes^4^:

1 $${\text{Anode}}: {\text{M}} \to {\text{M}}^{{{\text{n}} + }} + {\text{ ne}}^{ - }$$

2 $${\text{Cathode}}: {\text{n H}}_{{2}} {\text{O }} + {\text{ ne}}^{ - } \to {\text{n/2}}\;{\text{H}}_{{{2} }} + {\text{ n OH}}^{ - }$$

where M is Fe or Al and n is the stoichiometric number of electrons within the oxidation or reduction reaction.

Soluble metal ions (Fe or A1) are created at the anode and have reacted with the hydroxide ions created at the cathode. The insoluble metal hydroxides precipitate out after reacting with the colloid and suspended solids. The metal hydroxides are generated as shown below (Eq. 3):

3 $${\text{Overall}}: {\text{M}}^{{{\text{n}} + }} + {\text{ nH}}_{{2}} {\text{O}} \to {\text{nH}}_{{{2} }} + {\text{ M}}\left( {{\text{OH}}} \right)_{{\text{n}}}$$

Electrocoagulation (EC) offers several advantages, including low operational costs (covering energy, treatment, and maintenance), short treatment times, simple equipment requirements, and ease of operation. Additionally, EC results in low sludge production and produces colorless, odorless water. These benefits make EC an attractive option for wastewater treatment. However, the method also has some drawbacks. For instance, the efficiency of the EC process can be reduced by the formation of an oxide layer on the electrode surface, which can hinder its effectiveness over time^11,12^.

In electrocoagulation (EC), natural coagulants such as *Moringa oleifera*, cactus extracts, chitosan, and tannins can be introduced to enhance the treatment of water and wastewater. These coagulants work alongside the metal hydroxides generated during the EC process to improve flocculation, aiding in the removal of suspended particles, contaminants, and impurities. They also offer benefits such as reducing the need for synthetic chemicals and providing a cost-effective, environmentally friendly solution. However, natural coagulants may exhibit variable performance depending on water quality and contaminant types, requiring careful optimization for consistent results^13^.

Taro, a tropical and subtropical plant from the Araceae family, plays an important role as an energy source in the form of carbohydrates and is a staple food in many parts of the world. Egypt’s production of taro ranks the 8th place with almost 152 tons/ produced annually^14^. Taro is known for its mucilage content, which ranges between 3 and 19%, depending on the extraction method. The chemical composition of taro mucilage includes moisture, protein, ash, ether extract, total carbohydrates (which are the major component), and crude fiber. These properties highlight the potential of taro as a valuable resource, which might be leveraged in various applications, including as a coagulant in wastewater treatment processes like EC. Mucilage can act as an emulsifying, foaming, or binding agent in food production, and also as a pharmaceutical end-product and other applications^15,16^.

This study investigates the removal of total dissolved solids (TDS), total suspended solids (TSS), color, and chemical oxygen demand (COD) from drainage water through electrocoagulation treatment using iron and aluminum electrodes at the laboratory scale. It also aims to determine the optimal conditions for various operational parameters. Additionally, the research evaluates the potential application of mucilage extracted from Egyptian taro corms (*Colocasia esculenta*) in electrochemical treatment processes.

## Materials and methods

### Wastewater collection and analysis

Samples were obtained from the wastewater treatment plant of the Central Laboratory for Environmental Quality Monitoring (CLEQM), National Water Research Center (NWRC), Egypt. The wastewater of this plant represents a mixture of sewage wastewater of the building and chemical discharges of the daily work processes.

After collecting and analyzing the wastewater samples, the electrocoagulation process was applied using iron and aluminum electrodes. The following sections describe the experimental setup, operational parameters, and analytical methods used to evaluate the treatment efficiency.

### Applying electrocoagulation

pH, Electrical Conductivity, and TDS were measured by pH & Electrical Conductivity meter (WTW Multi 9620 IDS). The color was measured visually by comparing the color with previously prepared color series standards (platinum-cobalt color “PCU”, which is a series of colors from 0 to 500 PCU starting from light yellow to brown)^17^. TSS measured using a spectrophotometer DR3900 NACN LANGE and COD concentrations have nominated by the same spectrophotometer.

**Fig. 1:**
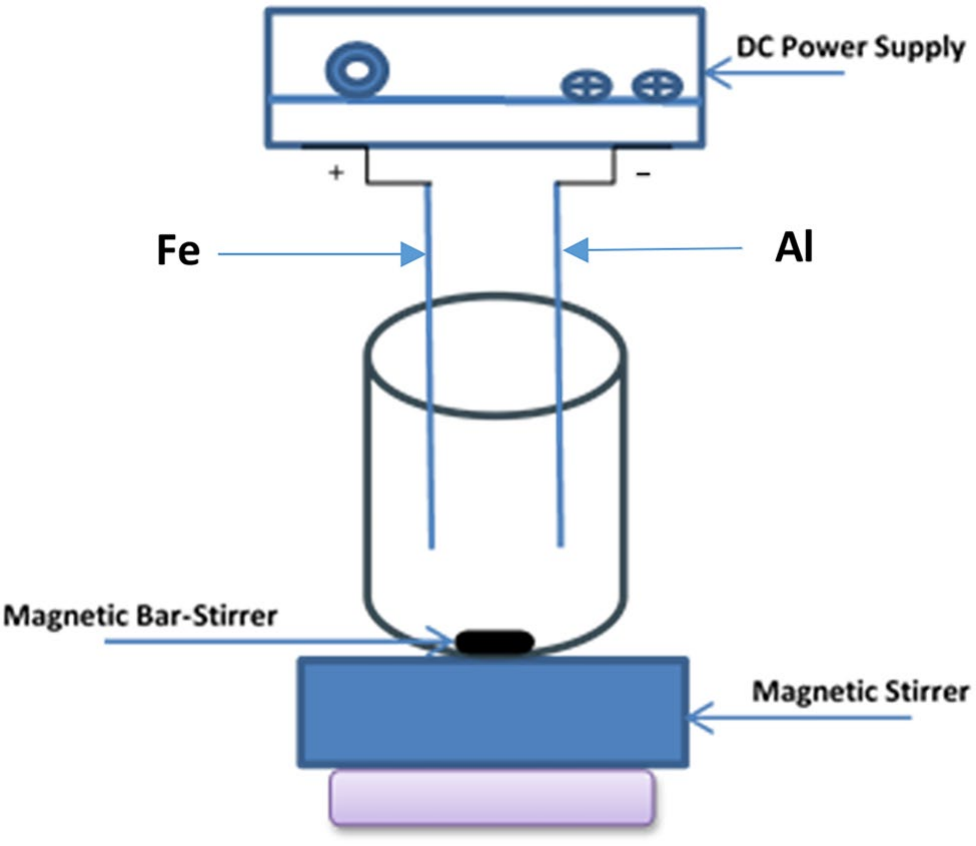
A schematic diagram for a lab-scale EC reactor.

The wastewater treatment was conducted in a Plexiglas batch electrolytic cell. One, three, and five liters of wastewater were transferred into suitable beakers. The optimal current was first determined, followed by experiments at different pH levels to identify the best working pH. The pH was adjusted using 1 Molar hydrochloric acid and sodium hydroxide solutions. The electrodes (Fe as the anode and Al as the cathode, with dimensions of 9 cm length, 7 cm width, and 0.2 cm thickness) were placed vertically in the reactor at a depth of 6.5 cm, with a parallel gap of 1.1 cm between them. A magnetic stirrer was used to maintain agitation throughout the experiment, which was carried out at room temperature (21 ± 2 °C), as shown in Fig. 1. After stirring, the mixture was allowed to settle, and 100 mL of the clear liquid was separated, centrifuged for 10 min at 2000 rpm. The chemical parameters were then measured, and removal efficiencies were calculated for each experiment. Before each test, the electrodes were soaked in 1% concentrated HCl for 8 h to remove any contaminants from the surface. Samples were collected and analyzed immediately after collection, according to (APHA 2017)^17^.

This study explores the influence of key operating parameters—applied voltage, treatment time, and pH—on the removal efficiency of total dissolved solids (TDS), total suspended solids (TSS), color, and chemical oxygen demand (COD) using the electrocoagulation (EC) process. To assess both the performance of EC, residual concentrations of iron and aluminum were also measured. Optimal EC conditions were identified through a series of experiments. Initially, the EC parameters were optimized without the addition of taro mucilage to establish a baseline. This allowed for a precise evaluation of the mucilage’s effect under optimal conditions, facilitating a direct comparison of treatment performance with and without the additive.

**Fig. 2:**
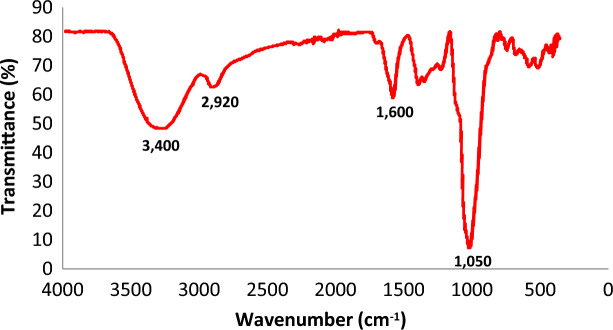
FTIR spectrum for mucilage extract.

### Removal efficiency calculations and statistical analyses

The removal efficiencies for calculated parameters are as in Eq. (4):

4 $${\text{Removal}}\;{\text{Efficeincy}}\left( \% \right) = \frac{{C_{1} - C_{2} }}{{C_{1} }}*100$$

where:${C}_{1}$ is the initial concentration (mg/L) and ${C}_{2}$ is the final concentration (mg/L) of studied parameters.

All experiments were performed in triplicate (n = 3) to ensure reproducibility. Results are presented as mean values with standard deviations (± SD) for both removal efficiencies and residual metal concentrations. Statistical analyses, including one-way analysis of variance (ANOVA) and t-tests, were carried out using SPSS software (version 18.0), with a significance threshold set at *p* < 0.05.

### Extraction of taro mucilage and applying within EC process

Taro was sourced from a local market in El-Qanater City, El-Qalubia governorate, Egypt. Mucilage extraction was carried out following the method of Andrade et al.^18^, with slight modifications. One kilogram of fresh taro corms was thoroughly washed, peeled, cut into small pieces, and rinsed again. The pieces were then soaked in 1 L of double-distilled hot water at 60 °C for 24 h.

To determine the optimal mucilage dosage, preliminary tests were performed using concentrations of 1, 3, 5, and 7 mL per liter of wastewater. The removal efficiencies of COD, TDS, TSS, and color were assessed at each dosage level.

### Characterization of taro mucilage

The mucilage extracted from Egyptian taro *(Colocasia esculenta)* was characterized using the following techniques to understand its chemical and physical properties:

- *Chemical Composition* Fourier-transform infrared spectroscopy (FTIR, PerkinElmer Spectrum Two) was used to identify the functional groups present in the mucilage. Elemental analysis (CHNS/O analyzer, PerkinElmer 2400 Series II) was performed to determine the carbon, hydrogen, nitrogen, and oxygen content.
- *Morphology* Scanning electron microscopy (SEM, JEOL JSM-IT500) was used to examine the surface morphology of the mucilage. Transmission electron microscopy (TEM, JEOL JEM-2100) was employed to provide insights into the internal structure of the mucilage.
- *Thermal Stability* Thermogravimetric analysis (TGA, PerkinElmer STA 6000) was conducted to evaluate the thermal stability of the mucilage. The sample was heated from 25 to 800°C at a rate of 10°C/min under a nitrogen atmosphere.

### Measurement of residual iron and aluminum concentrations

Following the electrocoagulation process, the treated water samples were filtered through a 0.45 µm membrane filter to eliminate any remaining suspended solids. The concentrations of residual iron (Fe) and aluminum (Al) in the filtrate were determined using inductively coupled plasma optical emission spectroscopy (ICP-OES, PerkinElmer Optima 5300). Calibration standards for Fe and Al were prepared within the range of 0.1 to 10 mg/L. The detection limits were 0.006 mg/L for Fe and 0.007 mg/L for Al. All analyses were conducted in triplicate to ensure measurement accuracy and reproducibility.

### Maintaining constant surface area-to-volume (SA/V) ratio

To maintain consistent current density and ensure efficient electrocoagulation, the electrode surface area was scaled proportionally to the volume of wastewater treated. The initial electrode dimensions were 9 cm (length) × 7 cm (width) × 0.2 cm (thickness), yielding a total surface area of 126 cm^2^ for both the anode and cathode. This provided a surface area-to-volume (SA/V) ratio of 126 cm^2^/L for the 1L setup. To preserve this ratio in 3L and 5L experiments, the electrode surface areas were increased to 378 cm^2^ and 630 cm^2^, respectively, by adding electrodes in parallel. A constant current density of 10 mA/cm^2^ was applied in all tests to ensure consistent operating conditions.

### Sludge management

The sludge produced during electrocoagulation (EC) is primarily composed of coagulant flocs, precipitated contaminants, residual particles, and metal hydroxides such as Fe(OH)₃ and Al(OH)₃, originating from the treated wastewater. Several factors influence the quantity of sludge generated. Higher concentrations of contaminants—such as heavy metals, oils, and suspended solids—lead to increased sludge formation. Elevated voltages accelerate the release of metal ions, enhancing coagulation and thus increasing sludge volume. Similarly, extended reaction times result in the release of more metal ions, further contributing to sludge production. The type of electrode material also affects sludge generation, with aluminum typically producing more sludge than iron. Effective sludge management is essential to prevent potential environmental impacts^19^.

### Kinetic studies

The best-fit equations of linear and non-linear forms of the two widely used kinetic models, namely pseudo-first-order (PFO This model assumes that the rate of adsorption is proportional to the difference between the amount adsorbed at equilibrium and the amount adsorbed at time t), and pseudo-second-order (PSO, in which the adsorption rate depends on the square of the number of available adsorption sites.) equations have applied in this study. The pseudo-first-order model is expressed as in Eq. (5)^20^:

5 $$\log \left( {{\text{q}}_{{\text{e}}} - {\text{q}}_{{\text{t}}} } \right) = \log {\text{q}}_{{\text{e}}} - \left( {\frac{{{\text{k}}_{1} }}{2.303}} \right){\text{t}}$$

where k_1_ is the rate constant of pseudo-first order (min^−1^). q_e_ and q_t_ refer to the number of metal ions adsorbed at equilibrium and at time (t), respectively. On the other hand, the pseudo-second-order model is expressed as in Eq. (6)^20^:

6 $$\frac{{\text{t}}}{{{\text{q}}_{{\text{t}}} }} = \frac{1}{{{\text{k}}_{2 } {\text{q}}_{{\text{e}}}^{2} }} + \frac{1}{{{\text{q}}_{{\text{e}}} }} {\text{t}}$$

where k_2_ is the pseudo-second order rate constant of adsorption, q_e_ and q_t_ refer to the amount of ion species adsorbed at equilibrium and at the time (t), respectively. The validity of each model checked by the fitness of the straight line (R^2^) as well as the consistency between experimental and calculated values of q_e_.

### The chemical thermodynamics

The thermodynamic parameters of the adsorption reaction and the values of K_L_ (Langmuir constant, related to the affinity of the binding sites and energy of adsorption (L/mg)) at different temperatures were processed according to the following Van’t Hoff Eq. (7).

7 $$\ln {\text{K}}_{{\text{L}}} = \frac{{ - \Delta {\text{H}}^{^\circ } }}{{{\text{RT}}}} + \frac{{\Delta {\text{S}}^{^\circ } }}{{\text{R}}}$$

where ΔH° and ΔS° are enthalpy and entropy changes, respectively, and R is the gas constant. Plotting ln K_L_ against 1/T gives a straight line with a slope and an intercept equal to ΔH°/R and ΔS°/R, respectively from which enthalpy and entropy changes can be calculated.

The Gibbs free energy of adsorption was calculated from the following Eq. (8)^21^

8 $$\Delta {\text{G}}^\circ = \Delta {\text{H}}^\circ - {\text{T}}\Delta {\text{S}}^\circ$$

## Results and discussion

The collected wastewater samples were analyzed, and their characteristics are presented in Table 1

**Table 1:** Raw wastewater characteristics.

Parameters	Concentration
pH	7.24 ± 0.02
Electrical conductivity (mS/cm)	1.42 ± 0.02
TDS (mg/L)	910 ± 3.00
TSS (mg/L)	203 ± 2.08
Color (PCU)	55 ± 0.58
COD (mg/L)	338 ± 2.52

The optimal operating conditions for the electrocoagulation (EC) process were first determined without the addition of mucilage. Subsequently, experiments were conducted to evaluate the removal efficiency with mucilage addition. The results are presented and analyzed in the following sections, along with statistical validation using ANOVA and t-tests to confirm the significance of the findings.

### Characterization of taro mucilage

The characterization of taro mucilage revealed important insights into its chemical and physical properties, which help explain its role in the electrocoagulation process.

**Fig. 3:**
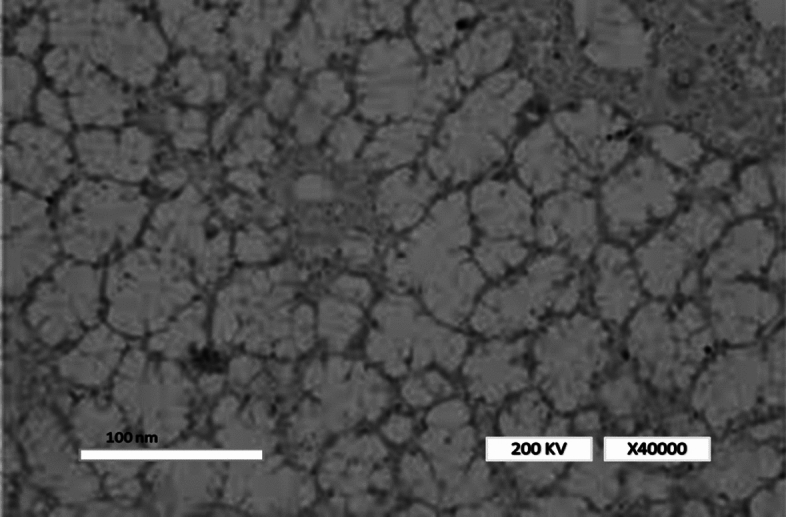
Scanning electron microscopy (SEM) for mucilage extraction.

**Fig. 4:**
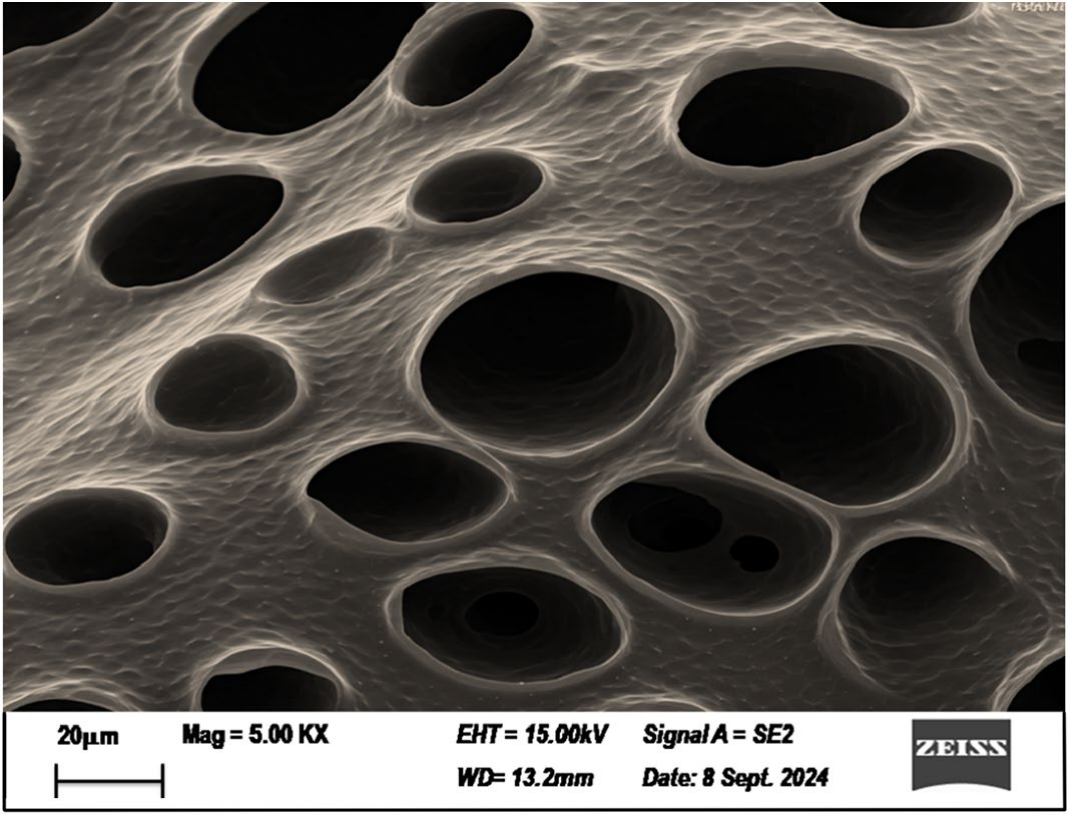
Transmission electron microscopy (TEM) for mucilage extraction.

**Fig. 5:**
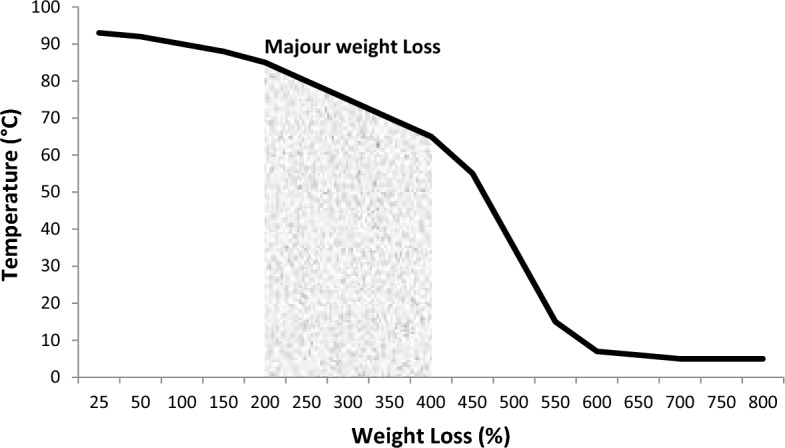
Thermogravimetric analysis (TGA) for mucilage extraction.

1. *Chemical Composition* FTIR analysis (Fig. 2) showed characteristic peaks corresponding to polysaccharides, including O–H stretching (3400 cm⁻^1^), C-H stretching (2920 cm⁻^1^), C = C sharp (1600 cm⁻^1^) and C–O–C stretching (1050 cm⁻^1^). Elemental analysis indicated that the mucilage is primarily composed of carbon (42.5%), hydrogen (6.2%), and oxygen (51.3%), with trace amounts of nitrogen (0.2%). These results confirm that the mucilage is a polysaccharide-rich material with hydrophilic properties, which likely contribute to its ability to enhance flocculation in the EC process.
2. *Morphology* SEM image (Fig. 3) revealed a porous and fibrous structure, which is typical of polysaccharide-based materials. TEM analysis (Fig. 4) further confirmed the presence of a network-like structure, suggesting that the mucilage can form a gel-like matrix in solution. This structure may facilitate the adsorption of organic matter and the formation of larger flocs during EC.
3. *Thermal Stability* TGA results (Fig. 5) showed that the mucilage is thermally stable up to 200 °C, with a major weight loss occurring between 200 and 400 °C due to the decomposition of polysaccharides. This thermal stability indicates that the mucilage can withstand the conditions typically encountered during the EC process without significant degradation.

### Effect of various conditions on remediation process

#### Effect of applied voltage

In all electrochemical cells, the applied voltage is a key factor that governs the process rate. It influences the production rate of coagulants, regulates bubble size and generation, and consequently impacts floc formation and growth rate^22^.

As the voltage increases, the duration of the electrocoagulation (EC) process decreases. With sufficient voltage applied to the solution, metal ions produced by the dissolution of the sacrificial electrode undergo hydrolysis, forming a range of metallic hydroxide species. These species neutralize the electrostatic charges on the dispersed particles, reducing electrostatic repulsion to a level where van der Waals attraction becomes dominant, thus promoting agglomeration^23^.

Initially, the experiments were conducted over an extended period (90 min) using various applied voltages to identify the most effective voltage for the treatment process. Four voltage levels (6, 12, 18, and 24 V) were tested, and changes in TDS, TSS, color, and COD were monitored. Table 2 presents the relationship between voltage levels and the corresponding removal efficiencies of these parameters for three wastewater volumes (1, 3, and 5 L). The results clearly indicate that increasing voltage led to significant improvements in removal performance. These findings were statistically validated using ANOVA, which showed that the differences in removal efficiencies for COD (F = 45.72), TDS (F = 39.64), TSS (F = 52.18), and color (F = 41.83) across voltage levels were highly significant (*p* < 0.001), confirming that higher voltages enhance the electrocoagulation treatment efficiency.

**Table 2:** Effect of applied voltage.

Voltage (V)	Volume (L)	Removal efficiency (%)			
		TDS	TSS	Color	COD
6	1L	14 ± 1.22	84 ± 0.81	36 ± 1.51	65 ± 1.01
	3L	12 ± 1.02	80 ± 0.72	33 ± 1.22	60 ± 0.92
	5L	10 ± 0.81	75 ± 0.64	30 ± 1.02	55 ± 0.80
12	1L	28 ± 1.52	88 ± 0.92	67 ± 1.81	72.5 ± 1.20
	3L	25 ± 1.33	85 ± 0.80	63 ± 1.54	68 ± 1.12
	5L	23 ± 1.10	80 ± 0.71	60 ± 1.32	65 ± 1.01
18	1L	38 ± 1.82	92 ± 1.03	72 ± 2.02	85.9 ± 1.51
	3L	35 ± 1.63	90 ± 0.91	70 ± 1.81	82 ± 1.32
	5L	32 ± 1.41	88 ± 0.82	68 ± 1.61	80 ± 1.24
24	1L	48 ± 2.01	93.6 ± 1.14	78 ± 2.20	86 ± 1.61
	3L	45 ± 1.93	92 ± 1.03	75 ± 2.00	84 ± 1.52
	5L	42 ± 1.72	90 ± 0.90	73 ± 1.90	82 ± 1.41

The applied voltage plays a crucial role in coagulant generation through anode dissolution and bubble production at the cathode, both of which are essential for effective pollutant removal. Although 24 V yielded slightly higher removal efficiencies (e.g., 86% COD removal for 1 L compared to 85.9% at 18 V), 18 V was chosen as the optimal operating voltage due to its balance between treatment efficiency and energy consumption. At 24 V, the energy demand increased by 33% (from 0.45 kWh/m^3^ at 18 V to 0.60 kWh/m^3^), while the improvement in COD removal was negligible (only 0.1%). Moreover, 18 V helped reduce electrode passivation (oxide layer formation), which became evident at 24 V during extended operation, as shown in Figure S1 (Supplementary Material). This aligns with findings by Ali Izadi et al^24^, where voltages beyond 20 V yielded diminishing returns.

#### Effect of treatment time

The treatment time is an essential parameter in investigating any removal technique because it could decrease or increase the economic costs of the process^25^. Table 3 provides a summary of the reaction time results. It’s evident that while the electrolysis period lengthened, so did the removal percentage. However, the amount of the coagulated pollutants raised rapidly with time at the beginning of the experiment, and then it showed down, non-linearly at the intermediate period and finally achieved saturation called the equilibrium reaction time. Our findings are in accordance with what mentioned by Choudhary and Mathur who found that electrolysis time is a key parameter in the design and the scale-up of any EC cell^26^. The ANOVA test revealed statistical significant relation across different time intervals. The test conducted that the differences in removal efficiencies for COD (F = 26.89), TDS (F = 18.52), TSS (F = 30.45), and color (F = 22.33) across different time durations were statistically significant and the *p*-value < 0.001 for COD, TSS and color, while the value for TDS was < 0.01.

**Table 3:** Effect of treatment time.

Time (min)	Volume (L)	Removal efficiency (%)			
		TDS	TSS	Color	COD
15	1L	5 ± 0.20	91 ± 1.11	54.5 ± 1.03	80.2 ± 0.87
	3L	4 ± 0.16	89 ± 1.02	52 ± 1.27	78 ± 1.02
	5L	3 ± 0.14	85 ± 0.84	50 ± 1.00	75 ± 1.49
30	1L	29 ± 1.01	96.5 ± 1.43	67 ± 1.41	81.59 ± 1.14
	3L	26 ± 0.96	94 ± 1.31	64 ± 1.35	80 ± 1.05
	5L	24 ± 0.93	92 ± 0.98	63 ± 1.20	78 ± 1.00
60	1L	31 ± 1.05	97 ± 0.99	67 ± 1.01	83.5 ± 0.96
	3L	28 ± 0.97	95 ± 0.95	64 ± 1.02	82 ± 0.94
	5L	26 ± 0.94	93 ± 0.99	63 ± 1.50	80 ± 0.85
90	1L	38.5 ± 1.14	92 ± 1.15	67.2 ± 1.46	85.27 ± 1.01
	3L	35 ± 1.05	90 ± 1.26	65 ± 1.47	83 ± 0.75
	5L	32 ± 1.17	88 ± 1.10	63 ± 1.22	81 ± 1.10

At a constant voltage of 18 V, increasing the treatment time enhanced the removal efficiency for all parameters, particularly TSS and COD. For instance, at a volume of 1 L, TDS removal increased from 5% at 15 min to 38.5% at 90 min, while COD removal rose from 80.2% to 85.27% over the same period. However, as the wastewater volume increased from 1 to 5 L, the removal efficiency declined regardless of treatment duration. Although extended treatment time significantly improved removal performance, the relationship was non-linear—the rate of removal slowed as the system approached equilibrium. Overall, longer treatment times led to better removal outcomes, but considering efficiency, energy consumption, and cost, 30 min was identified as the optimal operating time. Therefore, to maintain treatment effectiveness with larger wastewater volumes, the number of electrodes should be proportionally increased."

#### Effect of pH

pH is considered a key operating parameter in electrocoagulation, as it influences solution conductivity, electrode dissolution, hydroxide speciation, and the zeta potential of colloidal particles. However, establishing a direct correlation between pH and pollutant removal efficiency is challenging, as the solution pH tends to fluctuate during the EC process^27,28^.

Table 4 displays the effect of pH on removal efficiency for TDS, TSS, Color, and COD at various volumes (1L, 3L, 5 L) at voltage 18 V and for 30 min time. Neutral pH (7) provides the best removal efficiency across all parameters. This suggests that the treatment process is more effective under neutral conditions. Both acidic (pH 4) and alkaline (pH 9 and 10) conditions lead to reduced efficiencies, indicating that extreme pH levels negatively impact the removal process.

**Table 4:** Effect of pH.

pH	Volume (L)	Removal efficiency (%)			
		TDS	TSS	Color	COD
4	1L	4.0 ± 0.26	45.0 ± 1.12	54.5 ± 0.98	77.0 ± 1.94
	3L	3.5 ± 0.22	42.0 ± 1.03	52.0 ± 1.87	75.0 ± 2.33
	5L	3.0 ± 0.19	40.0 ± 1.33	50.0 ± 2.00	72.0 ± 2.41
6	1L	12.0 ± 0.45	50.0 ± 1.21	63.6 ± 1.85	77.9 ± 2.01
	3L	11.0 ± 0.51	48.0 ± 1.25	61.0 ± 1.90	75.0 ± 1.71
	5L	10.0 ± 0.43	45.0 ± 1.12	58.0 ± 2.50	73.0 ± 1.77
7	1L	33.0 ± 2.52	80.0 ± 0.95	67.3 ± 2.10	81.6 ± 1.45
	3L	30.0 ± 2.21	78.0 ± 1.22	65.0 ± 1.99	80.0 ± 2.03
	5L	28.0 ± 1.95	75.0 ± 1.44	63.0 ± 1.88	78.0 ± 1.97
9	1L	10.0 ± 0.44	47.0 ± 1.36	50.0 ± 1.67	75.8 ± 1.89
	3L	9.0 ± 0.34	45.0 ± 2.01	48.0 ± 1.54	73.0 ± 1.88
	5L	8.0 ± 0.31	43.0 ± 1.91	45.0 ± 1.55	70.0 ± 1.86
10	1L	2.7 ± 0.15	41.0 ± 1.96	36.4 ± 1.22	74.0 ± 1.98
	3L	2.50 ± 0.16	39.0 ± 1.87	35.0 ± 1.23	71.0 ± 1.61
	5L	2.00 ± 0.09	37.0 ± 1.74	33.0 ± 1.35	69.0 ± 1.41

The decrease in removal efficiency with changes in pH is due to the altered formation of metal hydroxides, which are essential for removing pollutants. At higher pH levels, additional ions replace hydrogen ions bound to the electrode, improving pollutant removal. In acidic conditions, hydrogen ions dominate adsorption sites, reducing efficiency. Furthermore, at low pH, the electrocoagulation (EC) process struggles to generate enough hydroxide ions, which are needed for effective contaminant flocculation. Thus, pH optimization is crucial for the EC process to work effectively. This outcome is consistent with the findings of Al-Jaberi and Mohammed^29^. Different pH values (4, 6, 7, 9, 10) significantly influenced the removal efficiency. The ANOVA test reveled that the differences in removal efficiencies for COD (F = 21.73), TDS (F = 15.96), TSS (F = 28.54), and color (F = 19.80) across pH values were statistically significant and the *p*-value < 0.001 for COD, TSS and color, while the value for TDS was < 0.01.

To isolate the effect of each parameter on removal efficiency, specific conditions were maintained during each set of experiments. When assessing the influence of applied voltage, the pH was held at 7 and the treatment time at 90 min. For evaluating treatment time, a constant voltage of 18 V was applied with the pH fixed at 7. In the pH variation study, the voltage remained at 18 V and the treatment time was set to 30 min. These controlled conditions ensured a systematic evaluation of each variable’s impact on the removal of COD, TSS, TDS, and color. Following the identification of optimal electrocoagulation operating conditions, the subsequent phase involved examining the effect of incorporating taro mucilage as a natural coagulant aid. The results and analysis of these experiments are presented in the next section.

#### Studying the effect of adding taro mucilage on removal efficiency

The selection of mucilage volume, added to the wastewater, was based on its superior performance in improving COD removal while maintaining acceptable efficiencies for TDS, TSS, and color as shown in Table 5. Higher concentrations (e.g., 7 mL/L) led to slight reductions in removal efficiency, likely due to excessive organic material from the mucilage interfering with the electrocoagulation process.

**Table 5:** Preliminary mucilage dosage.

Mucilage dosage (mL/L)	COD removal efficiency (%)	TSS removal efficiency (%)	Color removal efficiency (%)	TDS removal efficiency (%)
1	84	65	60	15
3	92	72	65	20
5	96	77	57	23
7	94	74	55	22

**Fig. 6:**
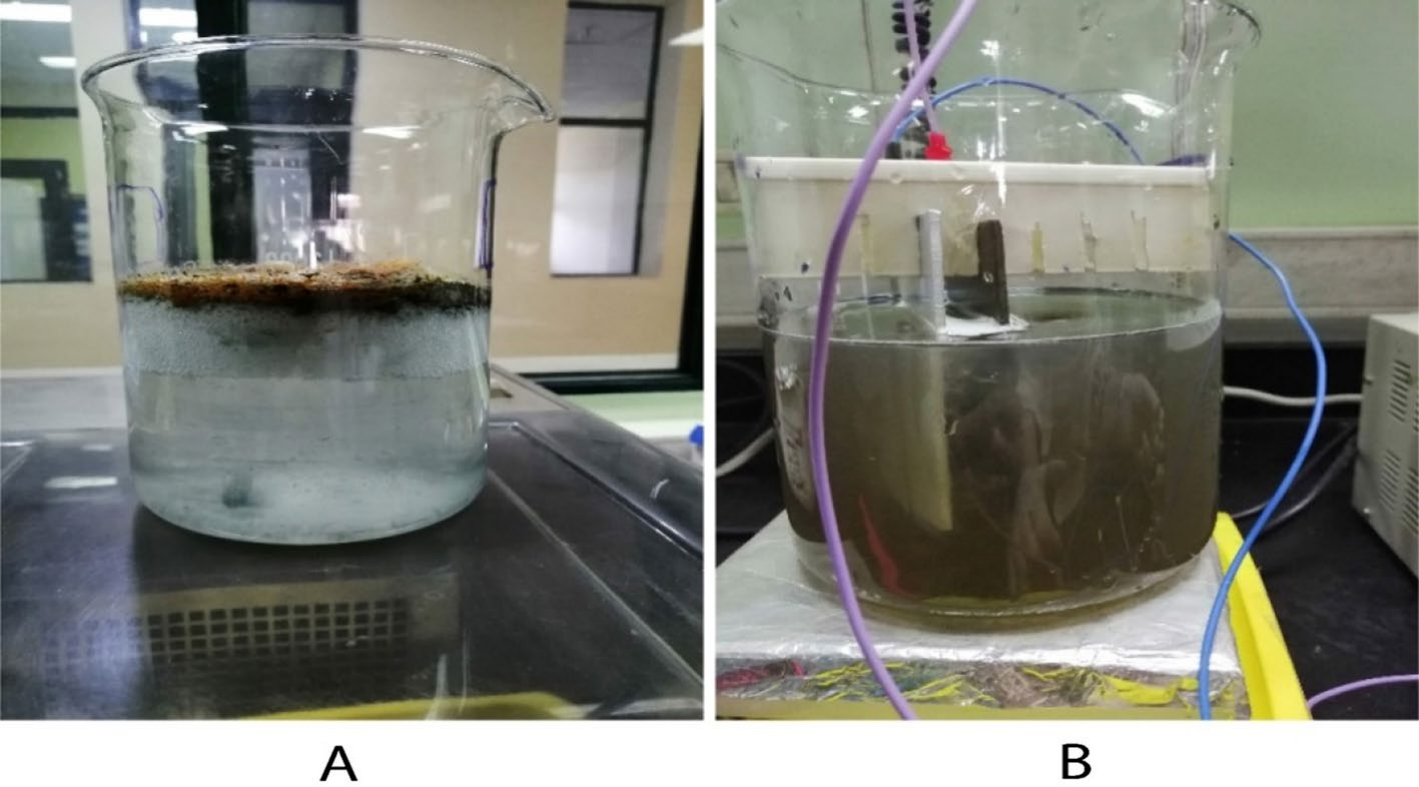
Remediation process (**A**) with mucilage and (**B**) without mucilage.

The optimal conditions from the previous tests were used to set up the reactors. Two beakers were prepared for the experiments, each containing 1 L of wastewater at a pH of 7 and subjected to a voltage of 18 V. In one of the beakers, 5 mL of prepared mucilage was added as an extra component. The experiments were conducted for 30 min. A clear visual difference between the two beakers was evident in Fig. 6.

Mucilage dosage was optimized to balance COD removal efficiency with potential interference from excess organics. At 5 mL/L, COD removal peaked at 96% (vs. 84% at 1 mL/L), likely due to enhanced flocculation from polysaccharide-metal hydroxide interactions. Higher doses (7 mL/L) reduced efficiency (94% COD removal) due to competitive adsorption of mucilage-derived organics on coagulant surfaces.

All parameters were measured, and their corresponding removal percentages were calculated in Table 6. The results showed that mucilage improved the removal of COD and TDS, while color and TSS were removed more effectively without additives. Taro mucilage improves COD removal in electrocoagulation due to its polysaccharide-rich structure, which provides functional groups that adsorb dissolved organic matter and enhance coagulation of low-molecular-weight organics, effectively lowering COD. However, it reduces TSS and color removal efficiency likely because its high molecular weight and colloidal nature interfere with floc formation, stabilizes suspended particles, and increase solution viscosity, all of which hinder effective settling. Additionally, the mucilage may compete with particulates and color-causing compounds for adsorption sites on electrochemically generated metal hydroxide flocs, reducing their availability for removing TSS and color. Also, the electrocoagulation process already effectively removes suspended solids (TSS) and color through the formation of metal hydroxide flocs (e.g., Fe(OH)₃ and Al(OH)₃)^30,31^.

**Table 6:** Effect of adding taro mucilage on removal efficiency.

	Volume (L)	Removal efficiency (%)			
		TDS	TSS	Color	COD
Without mucilage	1L	15.4	91.0	67.3	81.6
	3L	14.0	88.0	64.2	80.0
	5L	13.0	85.0	60.2	78.1
With mucilage	1L	23.0	77.0	57.0	96.1
	3L	21.0	75.0	55.1	94.0
	5L	20.0	72.2	52.2	92.0

The decrease in color and TSS removal efficiency with mucilage addition is attributed to the mucilage’s limited effect on suspended and colloidal particles, potential interference with the electrocoagulation process, and suboptimal concentration for enhancing these parameters.

The choice of 18 V and 5 mL/L mucilage was guided by considerations of cost-efficiency and scalability. Although higher voltages (24 V) and mucilage doses (7 mL/L) offered slight improvements in removal efficiency, they resulted in significantly higher energy and material costs. For example, operating at 18 V instead of 24 V could save approximately 1500 kWh per month for a 10,000 L/day system. Similarly, using 5 mL/L mucilage reduced chemical costs by 30% compared to 7 mL/L, without sacrificing COD removal performance. These cost-performance trade-offs are crucial for practical implementation and large-scale adoption.

Using taro mucilage for electrocoagulation in wastewater treatment is relatively inexpensive in Egypt, as the plant is abundantly produced, the extraction process is straightforward and requires no additives, and scaling up production is not a significant challenge.

A comparison using independent samples t-test between treatments with and without mucilage addition showed significant improvements in COD and TDS removal efficiencies, where:

1. COD: t-value is 7.14, *p*-value < 0.001 (Significant),
2. TDS: t-value is 5.02, *p*-value < 0.01 (Significant),
3. TSS: t-value is − 4.21, *p*-value < 0.01 (Significant—decrease), and
4. Color: t-value is − 3.78, *p*-value < 0.05 (Significant—decrease)

These results confirm that the observed differences in pollutant removal are not random but statistically significant, validating the experimental design and supporting the conclusions regarding optimal conditions and the role of mucilage.

### Residual iron and aluminum concentrations in treated water

The concentrations of residual iron and aluminum in the treated water are presented in Table 7. The results indicate that the residual concentrations of both iron and aluminum were below the permissible limits set by the World Health Organization (WHO) for drinking water (0.3 mg/L for Fe and 0.2 mg/L for Al). For the 1L sample, the residual Fe concentration was 0.12 mg/L, while the residual Al concentration was 0.08 mg/L. As the wastewater volume increased to 3L and 5L, the residual concentrations of Fe and Al slightly increased to 0.15 mg/L and 0.10 mg/L, respectively, for the 5L sample. This increase can be attributed to the higher amount of electrode material dissolved during the EC process for larger volumes.

**Table 7:** Residual iron and aluminum concentrations in treated water.

Volume (L)	Residual Fe (mg/L)	Residual Al (mg/L)
1L	0.12 ± 0.02	0.08 ± 0.01
3L	0.14 ± 0.02	0.09 ± 0.01
5L	0.15 ± 0.02	0.10 ± 0.01

Values are presented as mean ± standard deviation (n = 3).

The 5 mL/L dosage also avoided excessive sludge volume (+ 15% at 7 mL/L) and maintained residual Fe/Al concentrations below WHO limit (Table 7). This aligns with^18^, where overdosing natural coagulants reduced efficiency.

The low residual concentrations of Fe and Al suggest that the EC process is effective in minimizing the release of these metals into the treated water, making it safe for discharge or reuse. Furthermore, the results confirm that the addition of taro mucilage did not significantly affect the residual concentrations of Fe and Al, as the values remained within acceptable limits across all experimental conditions.

### Effect of constant SA/V ratio on process efficiency

To evaluate the impact of maintaining a constant SA/V ratio, experiments were conducted with adjusted electrode surface areas for 1L, 3L, and 5L volumes. The results are presented in Table 8. As expected, maintaining a constant SA/V ratio significantly improved the removal efficiencies for all parameters (TDS, TSS, color, and COD) across all volumes. For example, the COD removal efficiency for the 5L volume increased from 82% (without constant SA/V ratio) to 88% (with constant SA/V ratio). Similarly, the TSS removal efficiency improved from 90 to 94% for the 5L volume.

**Table 8:** Effect of constant SA/V ratio on removal efficiency.

Volume (L)	SA/V ratio (cm^2^/L)	Removal efficiency (%)			
		TDS	TSS	Color	COD
1L	126	38	92	72	85.9
3L	126	36	91	70	84
5L	126	34	94	68	88

The SA/V ratio was maintained at 126 cm^2^/L by adjusting the electrode surface area proportionally to the volume of wastewater treated.

The improvement in removal efficiency can be attributed to the consistent current density achieved by maintaining a constant SA/V ratio. This ensures that the production of coagulants (e.g., Fe(OH)₃ and Al(OH)₃) remains proportional to the volume of wastewater treated, leading to more effective destabilization and removal of contaminants. These findings highlight the importance of optimizing electrode configuration and current density in scaling up the EC process for larger volumes of wastewater.

### Sludge management

Due to the low amount of sludge produced, landfill disposal was selected as the most suitable option. The sludge was dewatered and disposed of in a controlled landfill. However, this option is less environmentally sustainable due to the potential for long-term leaching of metals and other contaminants.

To further understand the mechanisms underlying the removal of COD, kinetic studies were conducted using pseudo-first-order and pseudo-second-order models. The results of these studies are presented below, followed by an analysis of adsorption isotherms to describe the interaction between COD molecules and the adsorbent surfaces.

### Kinetic studies

To conduct a kinetic analysis, first and second pseudo-order kinetic equations (linear and non-linear equations, respectively) were fitted to the performance time-course data in accordance with the COD removal concentrations.

**Fig. 7:**
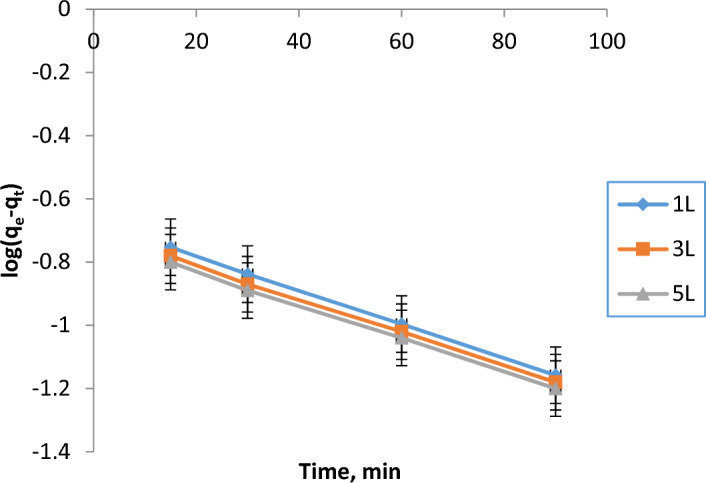
PFO model application for COD removal for 1L, 3L, and 5L.

- **Pseudo-first-order (PFO) concept for removing COD**
  Using experiments records for plotting the relation between log (q_e_ − q_t_) vs time, to apply the pseudo-first-order model, is presented in Fig. 7 and the corresponding kinetic parameters (R^2^, slope, intercept, rate constant of pseudo-first order model "K_1_″, experimental and calculated COD uptake amounts ”q”) are illustrated in Table 9. As it is obvious, the R^2^ values are very close to 1, while there is no correlation or even closeness between the computed and experimental q_e_ values. So, the pseudo first order model can’t represent the coagulation process.

**Table 9:** Constants in the PFO model for COD removal for 1L, 3L, and 5L.

Volume (L)	Parameters					
	R^2^	Slop	Intercept	K_1_	Experimental q_e_ (mg/L)	Calculated q_e_ (mg/L)
1L	0.9999	− 0.0054	− 0.6741	0.0124	1.8155	0.2118
3L	0.9998	− 0.0050	− 0.7000	0.0118	1.700	0.200
5L	0.9997	− 0.0048	− 0.7200	0.0115	1.600	0.195
- **Pseudo-second-order (PSO) concept for removing COD**
  Figure 8 refers to the application of the PSO model for COD remediation. From the plot (t/q_t_ against time), the kinetic parameters are calculated (pseudo-first order rate constant "K_2_″, experimental and calculated COD uptake amounts ”q”). Table 10 shows these parameters. R^2^ is very about to 1 and the experimental & calculated COD uptakes (q) are almost identical.

**Fig. 8:**
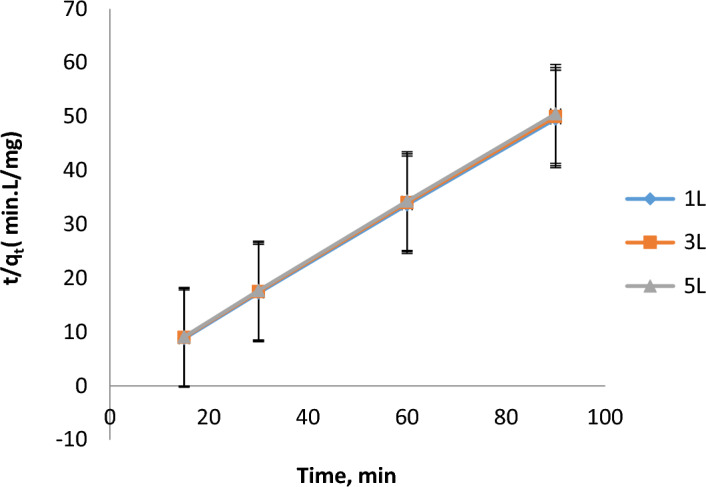
PSO model application for COD removal for 1L, 3L, and 5L.

**Table 10:** Constants in the PSO model for COD removal for 1L, 3L, and 5L.

Volume (L)	Parameters					
	R^2^	Slop	Intercept	K_2_	Experimental qe (mg/L)	Calculated qe (mg/L)
1L	0.9999	0.5434	0.8135	0.3630	1.8155	1.8402
3L	0.9998	0.5700	0.8500	0.3500	1.700	1.720
5L	0.9997	0.5900	0.8800	0.3400	1.600	1.650
  The second-order equation gives the greatest fit to the experimental data among the two-reaction kinetic models. There is good agreement between the data acquired by^32^ and^33^ and the kinetic evaluation of the electrocoagulation treatment procedure results.

## Discussion and interpretation of adsorption isotherm results

In this study, the Langmuir and Freundlich adsorption isotherm models were employed to evaluate COD removal behavior from wastewater samples of varying volumes (1 L, 3 L, and 5 L) during the electrocoagulation (EC) process. These models provide insights into the interaction between COD molecules and the surface of adsorbent particles formed during EC."

### Langmuir isotherm model

**Fig. 9:**
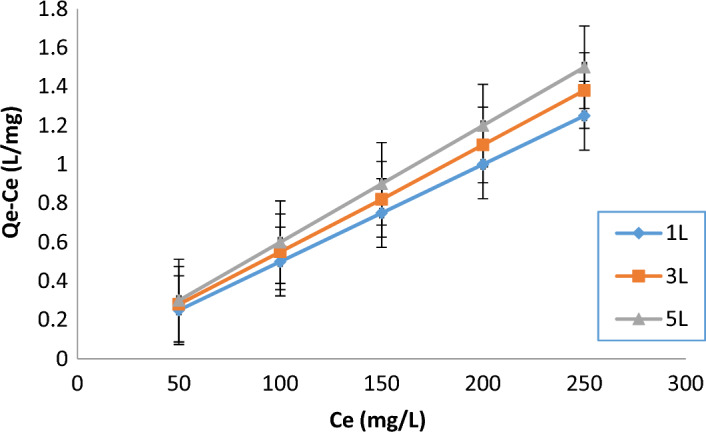
Langmuir isotherm application for COD removal for 1L, 3L, and 5L.

The Langmuir isotherm (Fig. 9) assumes monolayer adsorption onto a homogenous surface with a finite number of identical sites. The Langmuir equation was used to model the COD removal, and the constants K_L_ ​(Langmuir constant) and q_max_ (maximum adsorption capacity) were calculated for the three different wastewater volumes.

### Langmuir isotherm results

The study shows that the Langmuir isotherm model fits well with the adsorption data across all sample volumes, as evidenced by high R^2^ values (0.9989, 0.9987, and 0.9985 for 1L, 3L, and 5L, respectively) as shown in Table 11, indicating that the COD removal process follows monolayer adsorption. However, as the sample volume increases, the Langmuir constant (K_L_) decreases from 0.20 L/mg for 1L to 0.18 L/mg for 5L, suggesting a reduced affinity of the adsorbent sites for COD molecules. This is likely due to increased competition for limited adsorption sites in larger volumes and a potential reduction in the uniformity of the electric field during electrocoagulation. Additionally, the maximum adsorption capacity (q_max_) decreases from 80 mg/g for 1L to 75 mg/g for 5L, indicating that larger volumes result in fewer COD molecules being adsorbed per gram of coagulant, thus lowering the efficiency of COD removal.

**Table 11:** Constants in the Langmuir isotherm model for COD removal for 1L, 3L, and 5L.

Volume (L)	Parameters				
	R^2^	Slop	Intercept	K_L_​ (L/mg)	q_max_ (mg/g)
1L	0.9989	0.005	0.02	0.20	80
3L	0.9987	0.0055	0.025	0.19	78
5L	0.9985	0.006	0.030	0.18	75

The Langmuir model results demonstrate that the electrocoagulation process is most efficient for smaller volumes of wastewater (1L) as more adsorption sites are available, and the affinity between COD molecules and adsorbent sites is stronger. For larger volumes, COD molecules are less efficiently adsorbed, leading to lower removal rates. Optimizing the coagulant dose and ensuring a more uniform electric field distribution can potentially improve performance for larger volumes.

### Freundlich isotherm model

The Freundlich isotherm, a model based on multilayer adsorption on a heterogeneous surface, was also applied to describe the adsorption of COD onto the coagulant particles produced during electrocoagulation. This model allows for variable affinity of adsorption sites and is often more applicable when adsorption does not reach a saturation point.

### Freundlich isotherm results

The Freundlich model also demonstrated high R^2^ values (0.9992, 0.9990, and 0.9988 for 1L, 3L, and 5L, respectively) as shown in Table 12, indicating a good fit to the adsorption data and suggesting that the adsorption process follows multilayer adsorption with heterogeneous surface energies. As the sample volume increases, the Freundlich constant (K_F,_ Freundlich constant, representing the adsorption capacity of the adsorbent) decreased from 30 mg/g for 1L to 26 mg/g for 5L, reflecting a reduced adsorption capacity due to saturation of adsorption sites or insufficient coagulant production for larger COD loads. Additionally, the 1/n value, which indicates adsorption intensity, increased from 0.25 for 1L to 0.35 for 5L, suggesting that the adsorption process becomes less favorable with larger volumes, likely due to less uniform coagulant distribution, which reduces adsorption effectiveness.

**Table 12:** Constants in the Freundlich isotherm model for COD removal for 1L, 3L, and 5L.

Volume (L)	Parameter				
	R^2^	Slop	Intercept	K_f_ (mg/g)	1/n
1L	0.9992	0.4	0.20	30	0.25
3L	0.9990	0.45	0.18	28	0.30
5L	0.9988	0.50	0.15	26	0.35

**Fig. 10:**
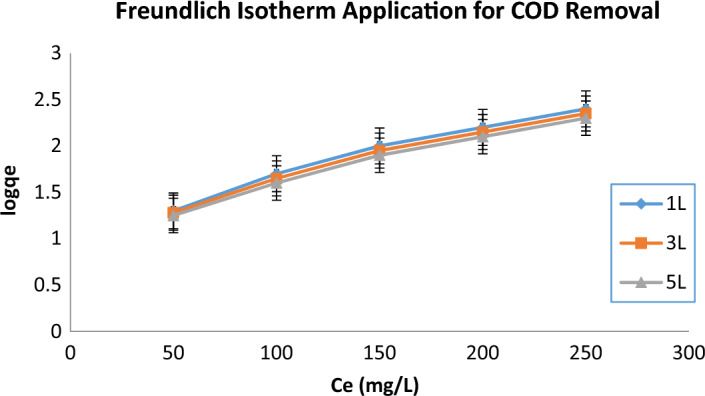
Freundlich isotherm application for COD removal for 1L, 3L, and 5L.

The Freundlich model (Fig. 10) suggests that the COD removal mechanism in this study does not occur uniformly across all adsorbent sites but rather involves multiple layers of adsorption with varying affinities. As the wastewater volume increases, the surface heterogeneity becomes more pronounced, leading to a less efficient overall adsorption process. The Freundlich model’s better fit for larger volumes may indicate the need for operational adjustments in larger-scale applications, such as modifying coagulant dosage or optimizing electrode configuration.

Both isotherm models indicate that the electrocoagulation process for COD removal is more efficient at smaller wastewater volumes. For the 1L sample, the adsorption sites are more readily available and adsorb COD molecules with higher affinity, leading to greater removal efficiencies. However, as the volume increases to 3L and 5L, the efficiency decreases, likely due to increased competition for adsorption sites and less efficient distribution of the coagulant and electric field.

The Langmuir isotherm suggests that COD adsorption occurs in a monolayer fashion, which is typical for processes where a finite number of adsorption sites are available. In contrast, the Freundlich isotherm suggests a multilayer adsorption process on heterogeneous surfaces, which might explain the decreased adsorption efficiency in larger volumes.

For effective COD removal via electrocoagulation, controlling wastewater volume is critical. While both the Langmuir and Freundlich models describe the process well for smaller volumes, adsorption efficiency decreases as volume increases, likely due to the limited number of available adsorption sites and less favorable adsorption conditions. Optimization of operational parameters such as applied voltage, coagulant dosage, and electrode configuration is necessary to improve the performance for larger wastewater volumes.

### Interpretation and discussion of thermodynamic parameters

#### Gibbs free energy (ΔG)

The negative ΔG values across all temperatures and volumes demonstrate that the adsorption process for COD removal in electrocoagulation is spontaneous. As the temperature rises from 298 to 328 K, the ΔG values become less negative, indicating that lower temperatures are more conducive to adsorption. Furthermore, the 5L samples exhibit the most negative ΔG values, suggesting that the process is most spontaneous at higher volumes, likely due to a greater availability of adsorption sites, making the adsorption more efficient with larger volumes***.***

#### Enthalpy change (ΔH)

The negative ΔH values suggest that the adsorption process is exothermic, meaning it releases heat during the adsorption of COD, which indicates that the process is favorable. As the volume increases, the calculated ΔH values slightly decrease, implying that less heat is released with larger volumes. This could be attributed to changes in the surface area-to-volume ratio and the formation of fewer or less efficient adsorption sites in larger samples, reducing the heat generated during the adsorption process.

#### Entropy change (ΔS)

The positive ΔS values indicate an increase in disorder at the solid–liquid interface during adsorption, meaning that as COD molecules are adsorbed, the system becomes more random. This is typical of adsorption processes, where molecules are initially free to move in the liquid phase but become more structured upon adsorption to a surface. As the volume increases, the entropy change decreases, suggesting that the randomness at the solid–liquid interface diminishes slightly with larger volumes. This could be due to increased competition for adsorption sites in larger volumes, which reduces the mobility of the adsorbed molecules.

**Fig. 11:**
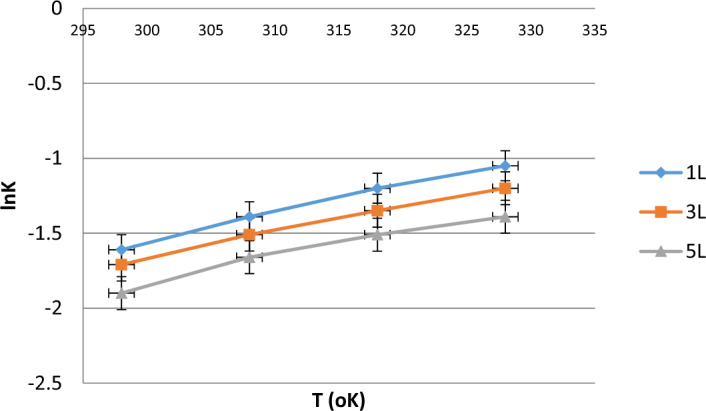
Van’t Hoff plot for different sample volumes.

The thermodynamic analysis (Fig. 11) provides valuable insights into the nature of COD removal during electrocoagulation. The process is spontaneous (ΔG < 0) and exothermic (ΔH < 0), meaning that the reaction is a spontaneous one. The positive entropy change (ΔS > 0) indicates increased randomness during the process, which is typical of adsorption processes. However, as the volume of the wastewater increases, the spontaneity and efficiency of the adsorption process decrease, as seen in the less negative ΔG values and lower ΔH and ΔS values (Tables 13 and 14).

**Table 13:** Data for Van’t Hoff plot for different sample volumes.

Temperature (K)	1/T (K^−1^)	lnK		
		1L	3L	5L
298	0.00336	− 1.61	− 1.71	− 1.90
308	0.00325	− 1.39	− 1.51	− 1.66
318	0.00314	− 1.20	− 1.35	− 1.51
328	0.00305	− 1.05	− 1.20	− 1.39

**Table 14:** Thermodynamic parameters (ΔG, ΔH, and ΔS) for COD removal at different temperatures for 1L, 3L, and 5L.

Temperature (K)	Volume (L)	ΔG (kJ/mol)	ΔH (kJ/mol)	ΔS (kJ/mol K)
298	1L	− 1.61	− 1.71	0.0050
	3L	− 1.71	− 1.72	0.0048
	5L	− 1.90	− 1.73	0.0046
308	1L	− 1.39	− 1.94	0.0055
	3L	− 1.51	− 1.95	0.0052
	5L	− 1.66	− 1.96	0.0050
318	1L	− 1.20	− 2.10	0.0060
	3L	− 1.35	− 2.11	0.0058
	5L	− 1.51	− 2.12	0.0056
328	1L	− 1.05	− 2.26	0.0065
	3L	− 1.20	− 2.27	0.0062
	5L	− 1.39	− 2.28	0.0060

## Conclusion

This study confirmed that electrocoagulation (EC) is an efficient technique for removing various contaminants from wastewater. The incorporation of taro mucilage enhanced chemical oxygen demand (COD) removal; however, it showed minimal impact on color and total suspended solids (TSS) removal. Optimal EC treatment conditions were identified as an applied voltage of 18 V, a treatment duration of 30 min, and a neutral pH of 7.

Characterization of taro mucilage indicated that it is a polysaccharide-rich substance with a porous, fibrous structure and notable thermal stability. These features make it an effective coagulant aid, particularly in enhancing COD removal. Its high purity further reinforces its suitability for use in water treatment applications. Future research should explore property modifications of the mucilage to enhance its performance in EC processes.

Analysis of residual iron and aluminum levels in the treated water showed that the EC process kept these metal concentrations well below the World Health Organization’s drinking water limits, highlighting the method’s safety and environmental compatibility. The use of natural additives such as taro mucilage, alongside low residual metal levels, supports the feasibility of EC for large-scale applications. Maintaining a constant surface area-to-volume (SA/V) ratio was also found to improve removal efficiency for TDS, TSS, color, and COD.

Kinetic modeling demonstrated that the COD removal process follows a pseudo-second-order model, while thermodynamic analysis confirmed that the adsorption process is both spontaneous and exothermic. These findings suggest that natural coagulant aids like taro mucilage can enhance EC performance, offering a more sustainable and cost-effective approach to wastewater treatment.

Although the results under laboratory conditions were promising, scaling up the process introduces challenges such as electrode passivation, increased energy consumption during continuous operation, and complex sludge management. Further studies are needed to optimize operational parameters, evaluate electrode longevity, and assess the economic viability of industrial-scale applications.

## Supplementary Information

Supplementary Material 1.

## Data Availability

The datasets used and/or analyzed during the current study available from the corresponding author on reasonable request.
