# Correction: Improving electrocoagulation performance by adding environmentally friendly materials

**DOI:** 10.1038/s41598-025-28165-5

**Published:** 2025-11-18

**Authors:** Manal M. M. Barakat, Mona S. S. Soliman, Mahmoud F. Mubarak

**Affiliations:** 1 Central Laboratory for Environmental Quality Monitoring (CLEQM), National Water Research Center (NWRC), El-Kanater, P.O. Box 13621/6, Qalubiya, Cairo Egypt; 2 Petroleum Application Department, Egyptian Petroleum Research Institute (EPRI), Cairo, 11727 Egypt; 3 Corelabcenter, Egyptian Petroleum Research Institute (EPRI), 1 Ahmed El Zomor St., Nasr City, Cairo 11727 Egypt

Correction to: *Scientific Reports* 10.1038/s41598-025-14462-6, published online 12 September 2025

The original version of this Article contained an error in equations 1 and 2, where

“bAnode: M → M^n+^ + ne^-^ (1)”

“Cathode: nH_2_O + ne^−^ → n/2H_2_ + n OH^-^ (2)”

now read:

1 $${\text{Anode:}}\quad {\text{M}} \to {{\text{M}}^{\text{n+}}} + {\text{n}}{{\text{e}}^-}$$

2 $${\text{Cathode:}}\quad {\text{n }}{{\text{H}}_{2}}{\text{O + n}}{{\text{e}}^{ -}} \to {\text{n}}/{{2}}\;{{\text{H}}_{{{2}}}} + {\text{n O}}{\text{H}}^{-}$$

Additionally, the Figure legends were swapped. Consequently, Fig. 2 legend,

“Remediation process (**A**) with mucilage and (**B**) without mucilage”

now reads:

“FTIR spectrum for Mucilage extract.”

Figure 3 legend,

“FTIR spectrum for mucilage extract”

now reads:

“Scanning Electron Microscopy (SEM) for Mucilage extraction.”

Figure 4 legend,

“Scanning electron microscopy (SEM) for mucilage extraction”

now reads:

“Transmission Electron Microscopy (TEM) for Mucilage extraction.”

Figure 5 legend,

“Transmission electron microscopy (TEM) for mucilage extraction.”

now reads:

“Thermogravimetric analysis (TGA)for Mucilage extraction.”

Figure 6 legend,

“Thermogravimetric analysis (TGA)for mucilage extraction”

now reads:

“Remediation Process (A) with mucilage and (B) without mucilage.”

Further, the Article contained an error in the Results and Discussion section, under the subheading ‘Characterization of taro mucilage’,

“2. *Morphology* SEM image (Fig. 4) revealed a porous and fibrous structure, which is typical of polysaccharide-based materials.”

now reads:

“2. *Morphology* SEM image (Fig. 3) revealed a porous and fibrous structure, which is typical of polysaccharide-based materials.”

And, under the subheading, ‘*Effect of various conditions on remediation process*’,

“A clear visual difference between the two beakers was evident in Fig. 2.”

now reads:

“A clear visual difference between the two beakers was evident in Fig. 6.”

Finally, the original Article contained an error in References, where Reference 18 was incorrectly cited. All sequential References were renumbered in the text.

“Effective sludge management is essential to prevent potential environmental impacts^18^”.

now reads:

“Effective sludge management is essential to prevent potential environmental impacts^19^”

The original Article has been corrected.

