# Supplementary material for: Improving electrocoagulation performance by adding environmentally friendly materials
Source: Sci Rep. 2025 Sep 12;15:32422. doi: 10.1038/s41598-025-14462-6 (PMC12432233; doi:10.1038/s41598-025-14462-6)
Supplement: Supplementary file 1 — Supplementary Material 1. [file 41598_2025_14462_MOESM1_ESM.docx]

**Table S1.** Energy Consumption vs. Voltage

| **Voltage (V)** | **COD Removal (%)** | **Energy (kWh/m³)** |
| --- | --- | --- |
| 12 | 72.5 | 0.30 |
| 18 | 85.9 | 0.45 |
| 24 | 86.0 | 0.60 |
